# Conservative management of haemorrhagic cholecystitis, haemobilia with obstructive jaundice: a case report

**DOI:** 10.1093/jscr/rjaf298

**Published:** 2025-07-21

**Authors:** Nikhil M Nagakumar, Vishal Lakhotia, Rushil Jain, Aditi Sachdeva, Sourav Panda, Manish Agarwal, Jagadeesh Krishnamurthy

**Affiliations:** Department of GI & Surgical Oncology, Max Smart Super Speciality Hospital, Saket, New Delhi 110017, India; Department of General Surgery, Max Super Speciality Hospital, Saket, New Delhi 110017, India; Department of General Surgery and Robotics, Max Super Speciality Hospital, Saket, New Delhi 110017, India; Department of General Surgery, Max Super Speciality Hospital, Saket, New Delhi 110017, India; Department of General Surgery, Max Super Speciality Hospital, Saket, New Delhi 110017, India; Department of General Surgery and Robotics, Max Super Speciality Hospital, Saket, New Delhi 110017, India; Centre for Liver and Biliary Sciences, Max Super Speciality Hospital, Saket, New Delhi 110017, India

**Keywords:** haemorrhagic cholecystitis, haemobilia, obstructive jaundice, intrahepatic gallbladder perforation, laparoscopic cholecystectomy

## Abstract

Haemorrhagic cholecystitis with haemobilia is a rare disease associated with high rates of morbidity and mortality if perforation and necrosis occur. Haemorrhagic cholecystitis and intrahepatic gallbladder perforation becomes hard to diagnose clinically and can be life threatening. This is a case of haemorrhagic cholecystitis, haemobilia with obstructive jaundice and intrahepatic gallbladder perforation in a COVID pneumonia patient. Emergency cholecystectomy in elderly (>70 yrs) is associated with high complication and mortality rates, therefore the decision whether or not to perform surgery should be well considered.

## Introduction

Gallstones can rarely present as haemorrhagic cholecystitis, which in turn can lead to haemobilia and obstructive jaundice. Haemorrhagic cholecystitis is a rare cause of haemobilia and an even more rare aetiology of biliary obstruction. Haemobilia refers to bleeding from or into the biliary tract, even though uncommon it’s an important cause of gastrointestinal bleeding [1]. We present a case of haemorrhagic cholecystitis, haemobilia with obstructive jaundice and intrahepatic gallbladder perforation in a COVID pneumonia patient under anticoagulation therapy, where the surgical risk-benefit profile favoured conservative approach.

## Case report

A 70-year-old COVID positive male patient with past medical history of type 2 diabetes mellitus, hypertension, gallstone disease and cerebrovascular accident (on clopidogrel and aspirin) presented to the emergency department with a chief complaint of pain abdomen for 10 days, increased in severity for 1 day. Associated symptoms included fever, vomiting, and black coloured stools. At presentation, he was febrile, pulse 108 beats/min and tenderness was present over the right upper quadrant, without any involuntary guarding or rebound tenderness.

The laboratory findings at the time of presentation were: total leucocyte count (TLC)-12 700/L; haemoglobin-8 g/dl; total serum bilirubin-5.1 mg/dl; aspartate transaminase (AST)-403 IU/L; alanine transaminase (ALT)-199 IU/L; alkaline phosphatase (ALP)-448 IU/L; gamma-glutamyl transpeptidase (GGT)-445 IU/L; serum lactate-1.8 mmol/L; activated partial thromboplastin time (APTT)-27.7 s and international normalized ratio (INR) was 1.38.

**Figure 1:**
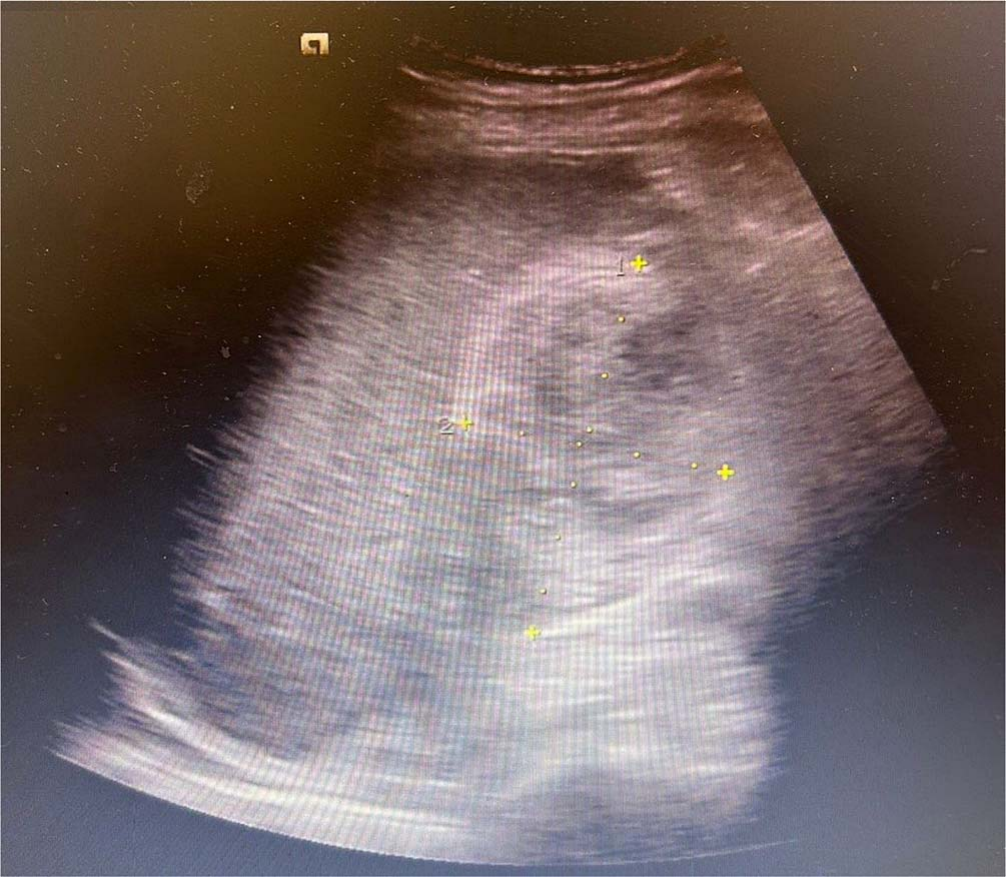
Ultrasound abdomen showing oedematous gallbladder with a heterogenous hypoechoic collection in gallbladder fossa (approximately measuring 9 × 4 cm).

Ultrasound abdomen showed oedematous gallbladder with a heterogenous hypoechoic collection in gallbladder fossa (approximately measuring 9 × 4 cm) (Fig. 1). Acute haemorrhagic cholecystitis and intrahepatic gallbladder perforation was confirmed with magnetic resonance cholangiopancreatography and CT angio abdomen (small contrast blush in gallbladder lumen).

Respiratory medicine team started injection remdesivir, injection methylprednisolone, 2 L of O2 supply via nasal prongs and other supportive measures for the COVID pneumonia management. Medical gastroenterology team took a conservative approach to manage haemobilia causing obstructive jaundice with a backup plan of endoscopic retrograde cholangiopancreatography if the liver function test worsens the next day. We, the surgical team decided to manage the patient conservatively looking at the surgical risk-benefit profile. He was started on IV antibiotics, tablet clopidogrel 75 mg/aspirin 150 mg was put on hold and was given 1 unit of packed red blood cells.

On hospital Day 2 and 3, the conservative management was continued as the laboratory values and the clinical condition of the patient did not worsen rather improved marginally. Patient even tested negative for COVID on Day 3.

On hospital Day 4, the clinical condition and laboratory values improved satisfactorily. Patient’s pulse rate came down to 80 beats/min and was relieved from pain and tenderness in right upper quadrant of the abdomen. His laboratory values improved overall with total bilirubin of 2.02, AST 60, ALT 83, ALP 320, GGT 286, HB 8.1, TLC 8.7, and serum lactate 0.5.

Patient was started on soft diet and oral anticoagulants were resumed on Day 5. Patient was discharged on Day 7 in stable condition with the advice of interval cholecystectomy. He was clinically well on the follow up visit and denied for interval cholecystectomy.

## Discussion

Haemorrhagic cholecystitis with haemobilia is a rare disease associated with high rates of morbidity and mortality if perforation and necrosis occur. The characteristic symptoms of haemobilia are abdominal pain, jaundice, and gastrointestinal bleeding through the common bile duct. As these symptoms resemble those of common hepatobiliary diseases, haemorrhagic cholecystitis can be easily missed by both physical and laboratory examinations. History taking, physical examination, laboratory findings, and imaging are important for the initiation of appropriate treatment of haemorrhagic cholecystitis.

Perforation of gallbladder is most often a rare complication of severe acute cholecystitis, but intrahepatic gallbladder perforation is extremely rare. Haemorrhagic cholecystitis and intrahepatic gallbladder perforation becomes hard to diagnose clinically and can be life threatening [2]. An early diagnosis can lead to good treatment outcomes [3]. Emergency cholecystectomy in elderly (>70 yrs) is associated with high complication and mortality rates [4], therefore the decision whether or not to perform surgery should be well considered.

In this present case, cholelithiasis and administration of oral anticoagulants seemed to be associated with the bleeding. Even though urgent surgical management (laparoscopic cholecystectomy) is now recommended in cholecystitis, according to surgeon experience, in order to prevent more serious complications [5], we considered conservative management in view of surgical risk-benefit profile favouring conservative approach.
